# Sugar feeding by invasive mosquito species on ornamental and wild plants

**DOI:** 10.1038/s41598-023-48089-2

**Published:** 2023-12-13

**Authors:** Irving Forde Upshur, Mikhyle Fehlman, Vansh Parikh, Clément Vinauger, Chloé Lahondère

**Affiliations:** 1 Department of Biochemistry, Virginia Polytechnic Institute and State University, Blacksburg, VA 24061 USA; 2 The Global Change Center, Virginia Polytechnic Institute and State University, Blacksburg, VA 24061 USA; 3 The Fralin Life Science Institute Virginia Polytechnic Institute and State University, Blacksburg, VA 24061 USA; 4 Center of Emerging, Zoonotic and Arthropod-Borne Pathogens, Virginia Polytechnic Institute and State University, Blacksburg, VA 24061 USA; 5 Department of Entomology, Virginia Polytechnic Institute and State University, Blacksburg, VA 24061 USA

**Keywords:** Entomology, Invasive species

## Abstract

Feeding on plant-derived sugars is an essential component of mosquito biology, affecting key aspects of their lives such as survival, metabolism, and reproduction. Among mosquitoes, *Aedes aegypti* and *Aedes albopictus* are two invasive mosquito species in the US, and are vectors of diseases such as dengue fever, chikungunya, and Zika. These species live in heavily populated, urban areas, where they have high accessibility to human hosts as well as to plants in backyards and public landscapes. However, the range of plants that are suitable sugar hosts for these species remains to be described, despite the importance of understanding what plants may attract or repel mosquitoes to inform citizens and municipal authorities accordingly. Here, we tested whether *Ae. aegypti* and *Ae. albopictus* would sugar-feed on eleven commonly planted ornamental plant species. We confirmed feeding activity using the anthrone method and identified the volatile composition of plant headspace using gas-chromatography mass-spectroscopy. These chemical analyses revealed that a broad range of olfactory cues are associated with plants that mosquitoes feed on. This prompted us to use plant DNA barcoding to identify plants that field-caught mosquitoes feed on. Altogether, results show that native and invasive mosquito species can exploit a broader range of plants than originally suspected, including wild and ornamental plants from different phyla throughout the Spring, Summer and Fall seasons.

## Introduction

Phytophagy, the act of feeding on plants, is important for many insect species, including blood-sucking mosquitoes. Acquiring carbohydrates is essential for both males and females and can, in some species, constitute the sole source of food for adults (*e.g.*, *Toxorhynchites* spp.). Males feed exclusively on plant-derived sugars and recently emerged females tend to seek for sugar before taking their first blood meal, which enhances egg production^1^. In addition to carbohydrates, it has been determined that mosquitoes also acquire vitamins, amino acids, and salts from plant nectar^2–4^.

To locate a sugar meal, mosquitoes are driven by several cues, including visual and olfactory (*e.g.*, plant-emitted semiochemicals)^5^. Volatile odorant compounds (VOCs) are perceived via odorant receptors present primarily on the antennae, which are fine-tuned to specific volatiles (either independently or in combination) and elicit attractive or repellent responses^6,7^. Terpenes and benzenoids are common compound classes in flower scent profiles that drive mosquito attraction. Some mosquito species have been shown to be attracted to flower semiochemicals like linalool, (Z)-3-hexen-1-ol, and benzaldehyde and repelled by compounds such as β-myrcene and limonene^5,8^. In addition, several studies have identified volatile compounds from fruits and plants that mosquitoes locate and feed on in the field^9,10^. However, a more comprehensive understanding of the semiochemicals associated with plants that are suitable sources of nectar to mosquitoes is critical to the development of new and efficient disease vector control tools, a pressing undertaking, as current control efforts are being challenged by increased insecticide resistance^11^. As an example, attractive toxic sugar baits (ATSBs) containing mixtures of attractive odorants and toxic compounds, take advantage of mosquitoes’ natural requirement to feed on sugar. Interestingly, these traps bypass preexisting problems that conventional control strategies have faced, such as insecticide resistance^12,13^. ATSBs have been successful in capturing several mosquito species, including *Aedes aegypti*, *Aedes albopictus*, *Aedes japonicus*, *Culex pipiens*, *Culex quinquefasciatus*, and *Anopheles gambiae*^14–19^.

Among disease vectors that are of particular concern are *Ae. aegypti* and *Ae. albopictus* mosquitoes. These species are responsible for spreading dengue, chikungunya and Zika viruses, all of which have high global incidence as vaccines and/or treatments remain unavailable^20^. According to a recent study by Leta et al.^20^, a total of 215 countries and territories exhibit environments that are suitable for *Ae. aegypti* and *Ae. albopictus* habitation. In the context of climate change and global warming, the geographic distribution of these mosquitoes might widen and potentially spread diseases in new areas, making it crucial to develop control strategies for both species^21^. This world-wide distribution further suggests that these species are capable of feeding on a broad range of plant hosts. However, their sugar feeding behavior is relatively understudied compared to their host-seeking and blood-feeding behaviors, primarily because pathogens are transmitted to humans and animals when an infected female bites a blood-host. Yet it appears crucial to study this behavior, as it bears the potential for the development of new tools for vector surveillance and control.

Resources that mosquitoes use in populated urban areas to obtain a sugar meal and how invasive species adapt to local ornamental plants remain poorly understood. Some ornamental plants (*e.g.*, *Ligustrum quihoui*, *Pittosporum tobira*, *Loropetalum chinense*) have been shown to increase survivorship in *Ae. albopictus* and could therefore contribute to their ability to successfully transmit pathogens by increasing their overall fitness^22^. Moreover, ornamental plant abundance has been shown to directly affect the population distributions of both *Ae. aegypti* and *Ae albopictus* during field experiments in Huixtla, Chiapas, Mexico for *Ae. aegypti* and Guangzhou, China and Long Island, New York, USA for *Ae. albopictus*^23–25^. Individuals from both species exhibited a higher proportion of sugar feeding when collected from an urban area with a higher amount of blooming ornamental plants. Altogether, this supports the hypothesis that ornamental species, by providing nectar to mosquitoes, might greatly influence their fitness and could consequently increase the risk of pathogen transmission in heavily populated areas. However, the breadth of ornamental plants that can serve as a source of carbohydrates to mosquitoes remains to be fully understood.

In this context, we first examined mosquito landing and feeding on different ornamental plant species that are commonly found in nurseries and backyards in the United States, with varying flower shape, size, color, scent, and nectar contents. We then analyzed the scent profile of each of these plants using gas-chromatography coupled with mass-spectrometry (GC–MS) to test whether attractive plants would share common chemical compounds. Because these results revealed that a broad range of chemical combinations are associated with suitable plant hosts, we used plant DNA barcoding on field-caught mosquitoes to identify plants that they are feeding on. Collectively, our results indicate that a broader than suspected range of plants can serve as a source of carbohydrates and the implications of these results for mosquito control are discussed herein.

## Results

### Plant visitation assays

We first selected 11 commercially available ornamental plants from a local nursery (Christiansburg, VA, USA) to determine whether mosquitoes would land and feed on some of the species, selected for their diversity in flower morphology and color: wave *Petunia*, red *Impatiens*, marigold, *Celosia*, butterfly bush, *Guara*, *Lantana,* Mexican heather, *Scaevola,* goldenrod and yarrow. We performed cage landing assays on three individual plants for each species and, independently, recorded the numbers of landings and feedings by males and females of *Ae. aegypti* and* Ae. albopictus.*

#### Landings

**Figure 1:**
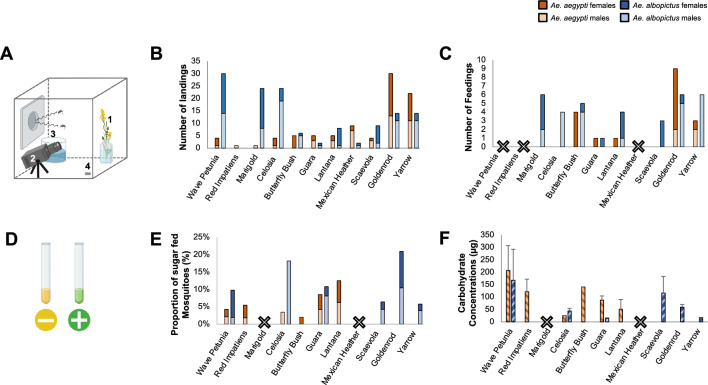
Mosquito sugar-feeding behavior on common ornamentals. (**A**) The standard layout for a plant visitation assay. Mosquitoes are released in a cage containing the ornamental flower [1], a GoPro aimed at the flower [2], a water cup topped with a soaked paper towel [3], and an iButton temperature/humidity recording device [4]. (**B**) Total number of observed landings by either *Ae. aegypti* males (light orange) and females (dark orange) or *Ae. albopictus* males (light blue) and females (dark blue) on the eleven different tested ornamental flower species (n = 3 × 20 mosquitoes (10M + 10F) per plant per mosquito species). (**C**) Total number of observed feedings. Ornamental flowers with a large ‘X’ indicate no activity from either species. (**D**) Negative (yellow) and positive (green) anthrone tests for the consumption of sugar. (**E**) Percentage of male and female *Ae. aegypti* and *Ae. albopictus* that tested positive for the consumption of fructose. (**F**) Total carbohydrate concentrations (µg) as determined by the anthrone method. Red impatiens and *Ae. albopictus* data is not present in these results (**E**, **F**), as an *Ae. albopictus* colony had not been established at the point of testing red impatiens.

When observed during a two-hour window centered on their spontaneous locomotor activity peak, *Ae. aegypti* visited 10 of the 11 plants tested, although low numbers of landings (< 6) were observed for 8 of these plants. We noted a higher number of visitations for the goldenrod and yarrow ornamental plant species (Fig. 1B; Generalized Linear Model: *p* = 0.0002 and *p* = 0.0027, respectively). Goldenrod had the highest number of landings from *Ae. aegypti*, while red impatiens and marigold had the least (one male “M” and one female “F”, respectively). Overall, we did not notice differences in landing activity between *Ae. aegypti* males and females (Generalized Linear Model: *p* = 0.7046), which is likely due to the low number of landings and number of replicates. Across all visitation assays, *Ae. aegypti* landed less on the plants compared to *Ae. albopictus,* although overall the mosquito species was not a significant predictor of the number of landings (Generalized Linear Mixed Model: *p* > 0.1). *Ae. albopictus* visited all of the 11 tested plants, but only 5 plant species received > 5 landings.

#### Feedings

By visual examination of the video recordings, we observed *Ae. aegypti* feeding on five ornamental plant species: butterfly bush, *Guara*, *Lantana*, goldenrod, and yarrow (Fig. 1C). Although the difference was not significant due to the low number of feeding events we observed (Generalized Linear Model: *p* = 0.101), female *Ae. aegypti* tended to feed more than males. We did not observe *Ae. aegypti* feeding on the wave petunia, red impatiens, marigold, *Celosia*, or *Scaevola,* which coincided with the low number of landings observed (Fig. 1B). On the other hand, *Ae. albopictus* fed on 8 out of the 11 tested plants, which coincides with the higher number of landings observed in this species. In contrast to *Ae. aegypti*, we observed a tendency for more total feeding events from male *Ae. albopictus* compared to females (Generalized Linear Model: *p* = 0.0594), although low sample sizes prevent us from concluding on sex differences. Finally, we did not observe any feedings from either mosquito species on Mexican heather, which corresponded with the low landing activity (Fig. 1B).

Looking at the proportion of visually-observed feeding events over the number of observed landings, we found that all plant species but the Mexican heather, *Scaevola*, and wave Petunia, were significant predictors of the proportion of feeding (Generalized Linear Model: *p* < 0.05). In *Ae. aegypti*, the proportion of feedings observed on butterfly bush was significantly higher than the next largest proportion of feedings observed on goldenrod (80% and 30%, respectively; Generalized Linear Model: *p* = 0.0065), and all the other plants *Ae. aegypti* fed on (Generalized Linear Model: *p* < 0.05). The butterfly bush was also the plant that elicited most feedings per landings in *Ae. albopictus*, and the proportion of feedings on this plant was significantly higher than on *Celosia*, goldenrod, *Guara* and *Lantana*, marigold, *Scaevola*, and yarrow (Generalized Linear Model: *p* < 0.0065).

#### Survival

Because the tested plants likely differ in the quality and quantity of carbohydrates available to mosquitoes, we next, assessed the impact of the sugar meal for each ornamental plant species. Specifically, we quantified mosquito survival following the plant visitation assays. Across the eleven ornamental species and for both *Ae. aegypti* and *Ae. albopictus,* females tended to exhibit higher average survival rates than males, although these differences were not statistically significant (Fig. S1A, B; Generalized Linear Model: *p* > 0.0893). In addition, the plant species was not a significant predictor of mosquito survival (Generalized Linear Model: *p* > 0.4152), which could either be due to the low sample size, or suggest that mosquitoes had ample energy reserves before the experiments.

### Carbohydrate content assays

Because mosquitoes may land and feed on plants outside of their peak activity time, we also used the anthrone colorimetric assay to identify successful sugar feedings that could have been missed by limiting our visual observations during mosquitoes’ peak activity time.

#### Qualitative analysis

First, the cold anthrone protocol was used to identify mosquitoes that were positive for fructose (Fig. 1E). Overall, *Ae. albopictus* exhibited a higher proportion of sugar feeding compared to *Ae. aegypti* (7.5% and 3.3%, respectively; Generalized Linear Model: *p* = 0.0028). No difference was observed between male and female *Ae. aegypti*, but male *Ae. albopictus* fed significantly more than females (11.9% and 4.45%, respectively; Generalized Linear Model: *p* = 0.0293). Overall, the plant species butterfly bush, *Celosia*, goldenrod, and *Guara* were significant predictors of the proportion of sugar feeding (Generalized Linear Model: *p* < 0.001, p = 0.033, *p* = 0.023, and *p* = 0.037). In addition, only *Ae. albopictus* tested positive for fructose when exposed to *Scaevola*, goldenrod, and yarrow, and only *Ae. aegypti* tested positive when exposed to butterfly bush and *Lantana*. No positive tests from either mosquito species were found for marigold and Mexican heather. It is worth noting that for some assays, such as goldenrod with *Ae. aegypti*, multiple feeding events were visually observed, but no mosquitoes tested positive for fructose consumption.

#### Quantitative analysis

The total amount of carbohydrates among the fructose-positive mosquitoes (n = 50) was quantified using the warm anthrone protocol (Fig. 1F). While the average sugar content of mosquitoes testing negative (n = 933) was below 30 µg (female *Ae. aegypti*: 16.9 ± 2.10 µg; male *Ae. aegypti*: 20.4 ± 5.51 µg; female *Ae. albopictus*: 9.95 ± 1.42 µg; male *Ae. albopictus*: 28.0 ± 13.7 µg), this amount increased by 1.43 folds in males and 11.15 folds in females *Ae. albopictus*, and by 3.28 folds in males and 6.62 folds in females *Ae. aegypti*. In negative-testing mosquitoes, no difference was found between species and sexes (Fig. S2A and S2B; Generalized Linear Model: *p* > 0.18). Overall, the sugar contents of positive-testing individuals of the two species were not significantly different (Generalized Linear Model: *p* = 0.3677), although carbohydrate concentrations tended to be higher in *Ae. aegypti* than *Ae. albopictus*. The plant species were not significant predictors of the mosquito sugar content either (Generalized Linear Model: *p* > 0.12), which could be explained by the low number of positive mosquitoes in our assays and likely due to the low number of replicates. Anecdotally, we found the highest carbohydrate concentration values in a female *Ae. albopictus* (541.43 µg ± 124.90 µg) and a male *Ae. aegypti* (301.43 µg ± 100.43 µg) that visited the wave petunia, followed by a female *Ae. aegypti* that visited *Lantana* (249.29 µg ± 39.71 µg).

### GC–MS analysis of plant odor

**Figure 2:**
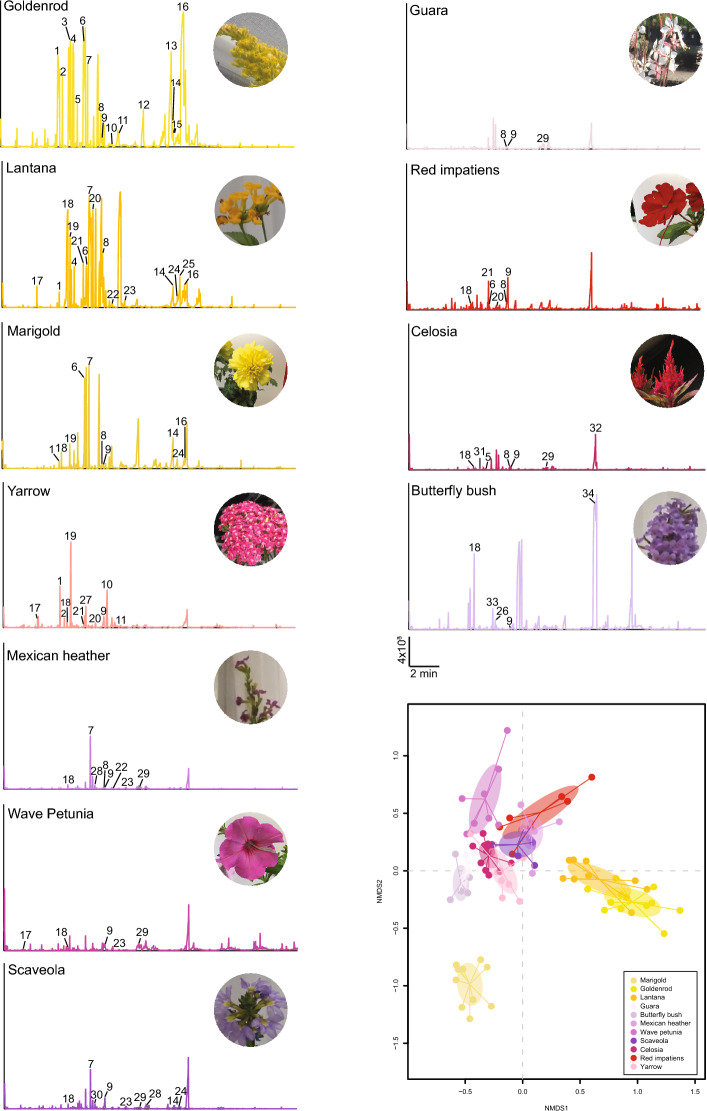
Example chromatograms for each of the eleven plants tested in the study. Pictures of the ornamental species are displayed on the right of each chromatogram. Scale: x-axis: au, arbitrary units, y-axis: time. Numbers above peaks within the chromatograms correspond to: 1, *ɑ*-pinene; 2, camphene; 3, *β*-pinene; 4, *β*-myrcene; 5, *ɑ*-phellandrene; 6, D-limonene; 7, *β*-ocimene; 8, linalool; 9, nonanal; 10, verbenol; 11, endo-borneol; 12, bornyl acetate; 13, aristolene; 14, caryophyllene; 15, γ-elemene; 16, germacrene D; 17, hexenal; 18, benzaldehyde; 19, *β*-phellandrene; 20, γ-terpinene; 21, p-cymene; 22, myroxide; 23, methyl salicylate; 24, *β-*farnesene; 25, humulene; 26, benzeneacetaldehyde; 27, eucalyptol; 28, m-ethylacetophenone; 29, p-cymen-7-ol; 30, γ-chlorobutyrophenone; 31, 5-octen-1-ol (Z); 32, *β-*bisabolene; 33, benzyl alcohol; 34, *ɑ*-farnesene. *Bottom right*: Nonmetric multidimensional scaling (NMDS) plot (stress = 0.1) of the chemical composition of the scent of all the plant species tested. Each dot represents a sample from a single individual plant. The ellipses represent the SD around the centroid of their respective cluster. Differences in scent composition and emission rate are significantly different between species (composition: ANOSIM, R = 0.93, *p* = 0.001).

To identify whether plants visited by mosquitoes shared common chemical cues, headspace collections and GC–MS analyses were conducted to resolve the VOC composition of the scent of each ornamental species (Fig. 2, Table 1). We found that terpenoids including linalool and aromatics such as nonanal were in abundance across each ornamental species tested. Nonanal had the highest relative concentration in the wave petunia (44.1%), red impatiens (35.7%) and *Guara* (57.8%). Other aliphatic compounds, including 2-hexanal and santolina triene, were present in small concentrations in all ornamental species except goldenrod. In *Lantana*, Mexican heather and *Scaevola*, *ß*-ocimene comprised the majority of the scent profile, representing 42.7%, 79.2%, and 56.9% of the scent composition, respectively. Benzaldehyde and limonene were found frequently across most of the ornamental species at varying concentrations. Benzaldehyde was found at its highest relative abundance in *Lantana* (29.6%), while limonene was found at high abundance in the scent of marigold (17.7%) and goldenrod (21.6%). *ß*-bisabolene was present only in the scent of *Celosia* but exhibited the highest relative peak abundance (30.8%). Germacrene D was a major constituent of the goldenrod scent (15%), and was present at smaller concentrations in marigold, *Lantana* and yarrow. We found that *ɑ*-pinene was in high abundance in the scent of goldenrod (25%) and was one of most abundant compounds in marigold (16.3%) and yarrow (7.7%). *ɑ*-farnesene was abundant only in the butterfly bush, making up 87.8% of the total scent’s peak area abundance. *ß*-phellandrene was found at small abundance in goldenrod, *Lantana,* and marigold, but had the highest relative peak abundance in yarrow (28%), with cis-verbenol being the second most abundant compound (26.8%). Finally, caryophyllene was the dominant compound in the scent profile of the marigold (20.5%) and was present at lower concentrations in *Scaevola* (8.4%) and yarrow (3.2%).

**Table 1:** Concentrations of ornamental volatile compounds.

	Wave Petunia	Red Impatiens	Marigold	Celosia	Butterfly Bush	Guara	Lantana	Mexican Heather	Scaevola	Goldenrod	Yarrow
Number of samples	7	6	5	4	5	7	10	9	7	11	7
Number of volatiles	14	13	22	18	15	17	28	18	17	27	28
Chemical name	Concentration [%]										
β-Acorenol	0.01 (± 0.01) [0.44%]	–	–	–	–	0.05 (± 0.02) [2.83%]	–	–	–	–	–
(-)-Aristolene	–	–	0.30 (± 0.22) [1.53%]	–	–	–	–	–	–	18.84 (± 7.04) [3.56%]	–
Benzaldehyde	0.15 (± 0.09) [4.95%]	0.01 (± 0.01) [0.45%]	0.35 (± 0.17) [1.74%]	0.05 (± 0.01) [0.94%]	0.30 (± 0.16) [1.51%]	–	55.94 (± 16.34) [29.61%]	0.09 (± 0.04) [0.88%]	0.02 (± 0.02) [0.36%]	0.08 (± 0.04) [0.02%]	0.16 (± 0.04) [1.05%]
Benzeneacetalaldehyde	–	–	0.50 (± 0.22) [2.49%]	–	0.02 (± 0.01) [0.13%]	–	–	–	–	–	–
Benzyl alcohol	–	–	–	–	0.16 (± 0.08) [0.80%]	–	0.20 (± 0.15) [0.10%]	–	–	–	–
trans-α-Bergamotene	–	–	–	–	0.06 (± 0.06) [0.29%]	–	0.31 (± 0.11) [0.15%]	0.02 (± 0.01) [0.17%]	0.06 (± 0.03) [1.51%]	–	–
β-Bisabolene	–	–	–	1.58 (± 0.23) [30.80%]	–	–	–	–	–	–	–
endo-Borneol	–	–	–	–	–	–	–	–	–	2.45 (± 0.62) [0.48%]	0.20 (± 0.07) [1.36%]
Bornyl acetate	–	–	–	–	–	–	–	–	–	8.94 (± 1.74) [1.76%]	–
Camphene	–	–	–	–	–	–	–	–	–	6.99 (± 0.94) [1.36%]	0.21 (± 0.11) [1.42%]
Camphor	–	–	0.01 (± 0.01) [0.06%]	–	0.01 (± 0.01) [0.04%]	–	–	–	0.01 (± 0.01) [0.21%]	–	0.24 (± 0.10) [1.58%]
(+)-4-Carene	–	–	–	–	–	0.03 (± 0.03) [1.84%]	0.36 (± 0.1) [0.20%]	–	–	0.59 (± 0.11) [0.11%]	0.10 (± 0.05) [0.63%]
Caryophyllene	–	–	4.10 (± 0.77) [20.65%]	–	–	–	4.66 (± 1.5) [2.58%]	0.20 (± 0.13) [1.92%]	0.35 (± 0.12) [8.35%]	1.98 (± 1.42) [0.38%]	0.47 (± 0.20) [3.15%]
Cedrene	0.06 (± 0.01) [2.09%]	–	–	0.03 (± 0.01) [0.61%]	–	0.03 (± 0.01) [1.98%]	0.09 (± 0.02) [0.04%]	–	0.03 (± 0.01) [0.66%]	0.21 (± 0.05) [0.04%]	0.10 (± 0.02) [0.39%]
Chlorobutyrophenone	–	–	–	0.02 (± 0.01) [0.41%]	–	0.02 (± 0.01) [1.09%]	0.02 (± 0.01) [0.01%]	–	0.09 (± 0.05) [2.16%]	–	–
Citral	0.01 (± 0.01) [0.31%]	–	0.36 (± 0.16) [1.82%]	–	–	–	0.19 (± 0.07) [0.12%]	–	–	0.23 (± 0.01) [0.04%]	–
o-Cymene	–	0.35 (± 0.05) [15.76%]	–	–	–	–	2.7 (± 0.64) [1.49%]	–	–	0.36 (± 0.07) [0.07%]	0.08 (± 0.05) [0.53%]
p-Cymen-7-ol	0.39 (± 0.03) [12.57%]	0.05 (± 0.05) [2.25%]	0.19 (± 0.04) [0.97%]	1.40 (± 1.15) [27.29%]	0.05 (± 0.05) [0.23%]	0.18 (± 0.12) [10.77%]	0.53 (± 0.17) [0.32%]	0.15 (± 0.06) [1.51%]	0.17 (± 0.06) [3.99%]	–	0.09 (± 0.07) [0.57%]
γ-Elemene	–	-	–	–	–	0.01 (± 0.01) [0.14%]	–	0.01 (± 0.01) [0.06%]	–	7.76 (± 6.08) [1.37%]	–
m-Ethylacetophenone	–	–	0.21 (± 0.09) [1.01%]	0.04 (± 0.01) [0.86%]	–	–	0.85 (± 0.31) [0.52%]	0.06 (± 0.06) [0.63%]	0.21 (± 0.07) [4.84%]	0.09 (± 0.06) [0.02%]	0.07 (± 0.07) [0.40%]
Eucalyptol	–	–	–	–	–	–	–	–	–	–	1.09 (± 0.27) [7.20%]
α-Farnasene	–	–	–	0.01 (± 0.01) [0.17%]	17.69 (± 10.82) [87.83%]	–	–	0.01 (± 0.01) [0.06%]	0.01 (± 0.01) [0.24%]	–	0.02 (± 0.01) [0.13%]
cis-β-Farnasene	–	–	0.03 (± 0.01) [0.17%]	0.05 (± 0.01) [0.97%]	0.01 (± 0.01) [0.02%]	0.01 (± 0.01) [0.47%]	1.80 (± 0.86) [1.01%]	0.03 (± 0.02) [0.28%]	0.09 (± 0.03) [2.04%]	–	–
a,d-Gala-octonic*	–	–	–	–	–	0.04 (± 0.02) [2.24%]	–	–	–	–	–
Germacrene D	–	–	0.75 (± 0.17) [3.77%]	–	–	–	4.49 (± 1.5) [2.50%]	–	–	77.48 (± 11.29) [15.01%]	0.24 (± 0.17) [1.43%]
α-Guaiene	–	–	0.88 (± 0.20) [4.44%]	–	–	–	–	–	–	–	–
Heptacosane	0.49 (±0.19) [15.92%]	–	–	–	–	–	–	–	–	–	–
2-Hexenal	0.05 (± 0.02) [1.67%]	0.08 (± 0.03) [3.79%]	0.09 (± 0.03) [0.44%]	0.04 (± 0.03) [0.98%]	0.04 (± 0.02) [0.18%]	0.04 (± 0.02) [2.42%]	0.31 (± 0.19) [0.17%]	–	–	–	0.34 (± 0.17) [2.41%]
Humulene	–	–	–	–	–	–	7.10 (± 2.4) [3.93%]	–	–	–	–
d-Limonene	–	0.11 (± 0.11) [5.21%]	3.53 (± 0.63) [17.82%]	–	–	–	0.24 (± 0.16) [0.13%]	–	–	110.93 (± 17.24) [21.61%]	–
Linalool	–	0.24 (± 0.18) [11.07%]	0.29 (± 0.07) [1.35%]	1.04 (± 0.61) [20.28%]	0.06 (± 0.06) [0.32%]	0.18 (± 0.06) [10.73%]	7.91 (± 2.92) [4.05%]	0.22 (± 0.07) [2.13%]	0.04 (± 0.04) [1.04%]	3.92 (± 0.83) [0.77%]	0.06 (± 0.06) [0.41%]
Methyl Salicylate	0.20 (± 0.05) [6.50%]	–	0.13 (± 0.03) [0.65%]	–	–	–	2.59 (± 0.55) [1.46%]	0.15 (± 0.10) [1.29%]	0.08 (± 0.05) [1.91%]	0.29 (± 0.06) [0.06%]	0.06 (± 0.03) [0.40%]
γ-Muurolene	–	–	–	–	–	0.01 (± 0.01) [0.53%]	0.25 (± 0.13) [0.13%]	–	0.01 (± 0.01) [0.17%]	1.11 (± 0.22) [0.22%]	–
β-Myrcene	–	–	–	–	–	–	4.40 (± 0.63) [2.49%]	–	–	54.44 (± 12.87) [10.59%]	–
Myroxide	–	–	1.24 (± 0.57) [6.28%]	–	–	–	1.00 (± 0.19) [0.56%]	0.06 (± 0.04) [0.54%]	–	–	–
Naphthalene	0.07 (± 0.01) [2.17%]	0.06 (± 0.01) [2.63%]	–	–	–	0.03 (± 0.01) [1.90%]	–	0.04 (± 0.01) [0.39%]	–	–	–
Nerolidol	–	–	–	0.01 (± 0.01) [0.09%]	0.05 (± 0.02) [0.24%]	–	0.03 (± 0.03) [0.01%]	0.06 (± 0.04) [0.58%]	0.01 (± 0.01) [0.24%]	–	0.10 (± 0.04) [0.68%]
Nonanal	1.37 (± 0.41) [44.07%]	0.79 (± 0.19) [35.73%]	0.57 (± 0.11) [2.85%]	0.69 (± 0.04) [13.47%]	1.45 (± 0.84) [7.17%]	0.95 (± 0.19) [57.77%]	0.60 (± 0.60) [0.31%]	0.82 (± 0.11) [8.05%]	0.60 (± 0.17) [14.14%]	2.58 (± 0.34) [0.51%]	1.53 (± 0.47) [9.85%]
6-Nonenal	–	–	–	–	0.07 (± 0.03) [0.37%]	0.01 (± 0.01) [0.44%]	–	–	–	–	0.09 (± 0.04) [0.60%]
1-Nonen-3-ol	0.01 (± 0.01) [0.03%]	–	–	–	0.13 (± 0.06) [0.67%]	–	–	–	–	–	–
β-Ocimene	–	–	2.87 (± 0.99) [14.49%]	–	–	–	82.08 (± 27.85) [42.72%]	8.07 (± 1.18) [79.24%]	2.47 (± 0.81) [56.93%]	5.53 (± 1.15) [1.07%]	0.16 (± 0.16) [0.95%]
Octanal	–	–	–	–	–	–	–	–	–	–	0.10 (± 0.06) [0.64%]
5-Octen-1-ol (Z)	–	–	–	0.03 (± 0.01) [0.55%]	–	0.01 (± 0.01) [0.84%]	–	0.01 (± 0.01) [0.03%]	–	–	–
Pentacosane	0.07 (± 0.02) [2.28%]	–	–	–	–	–	–	–	–	–	–
α-Phellandrene	–	–	–	0.06 (± 0.03) [1.11%]	–	–	–	–	–	2.80 (± 0.47) [0.55%]	0.02 (± 0.02) [0.16%]
β-Phellandrene	–	–	0.07 (± 0.07) [0.37%]	–	–	–	0.67 (± 0.46) [0.50%]	–	–	–	4.31 (± 2.76) [28.00%]
α-Pinene	–	–	3.23 (± 0.93) [16.26%]	–	–	–	1.30 (± 0.33) [0.80%]	–	–	130.03 (± 34.02) [24.98%]	1.15 (± 0.45) [7.65%]
β-Pinene	–	–	0.06 (± 0.06) [0.31%]	–	–	–	–	–	–	16.84 (± 9.83) [3.36%]	–
Sabinene	–	–	–	–	–	–	–	–	–	50.34 (± 8.29) [9.80%]	–
Santolina triene	0.17 (± 0.03) [5.45%]	0.09 (± 0.02) [4.12%]	–	–	0.03 (± 0.02) [0.15%]	0.05 (± 0.02) [3.06%]	–	0.07 (± 0.02) [0.73%]	0.04 (± 0.02) [0.99%]	–	–
γ-Terpinene	–	0.34 (± 0.13) [15.63%]	–	–	–	–	6.23 (± 1.53) [3.57%]	–	–	0.69 (± 0.17) [0.14%]	0.10 (± 0.06) [0.67%]
α-Terpineol	–	0.04 (± 0.01) [1.72%]	0.09 (± 0.05) [0.46%]	0.03 (± 0.01) [0.62%]	–	0.01 (± 0.01) [0.60%]	–	0.04 (± 0.03) [0.41%]	–	7.82 (± 4.11) [1.54%]	0.02 (± 0.01) [0.15%]
α-Thujenal	–	0.01 (± 0.01) [0.54%]	–	0.01 (± 0.01) [0.09%]	–	–	–	–	–	–	0.01 (± 0.01) [0.11%]
Tumerone	0.04 (± 0.03) [1.31%]	0.02 (± 0.02) [1.10%]	–	0.03 (± 0.02) [0.52%]	–	–	–	–	–	–	–
cis-Verbenol	–	–	–	0.01 (± 0.01) [0.25%]	–	–	0.13 (± 0.03) [0.07%]	–	–	1.02 (± 0.36) [0.20%]	4.05 (± 1.34) [26.84%]

Concentrations are represented as an average in ng/µL. The proportion of each compound relative to other compounds present in each scent is displayed as a percentage. *a,d-Gala-octonic phenylhydrazide.

We conducted an NMDS (non-metric multidimensional scaling) analysis to compare the scent profiles of each ornamental plant species based on the chemical compounds and their relative abundance (Fig. 2). Most of the ornamental species exhibited a distinct clustering of samples, indicating a unique and distinct scent composition (ANOSIM, R = 0.93, *p* = 0.001) (stress = 0.1), despite the aforementioned overlap in certain chemicals. Of note, marigold, *Lantana* and goldenrod samples showed individual clustering and separated from the main cluster. The scent profile of these three plants contains volatile compounds that are highly abundant relative to other ornamentals and, interestingly, goldenrod and *Lantana* samples clustered close together, while the marigold samples were isolated in the bottom-left quadrant of the NMDS. This positioning also reflects the behavior of the mosquitoes as neither species obtained carbohydrates from marigold.

Though the chemical profiles of plant scents are not enough to predict mosquito feeding behavior on their own, the broad range of chemical signatures associated with plants that were visited and fed on by mosquitoes suggests that an even broader range of plants than suspected could serve as sources of carbohydrates in the wild.

### Plant DNA barcoding

The aforementioned laboratory experiments identified commercially available ornamental plants that can be visited and fed upon by mosquitoes. However, these plants' short seasonal blooming windows did not allow a wide coverage of the full range of plants potentially available to mosquitoes in the field. In addition, the breadth of chemical signatures found in plant hosts suggests an even broader range of suitable plant hosts. To circumvent limitations associated with the laboratory-based testing of individual plants, we relied on plant DNA barcoding to identify the plant species that field-caught mosquitoes fed upon.

#### Carbohydrate analysis

**Figure 3:**
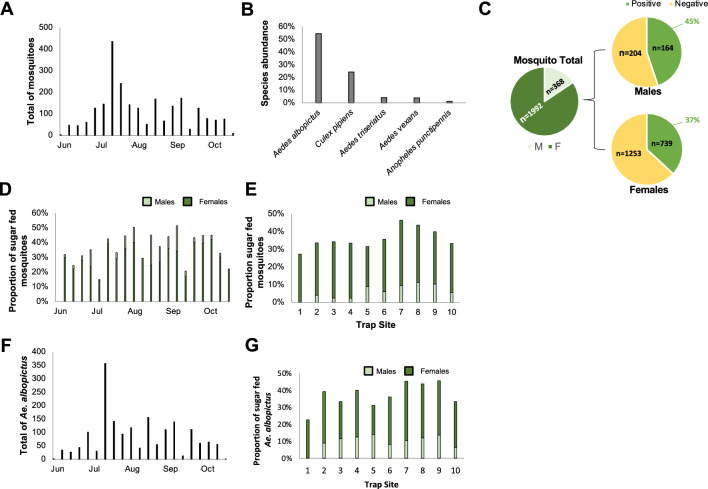
Mosquito sugar-feeding activity in urban areas. (**A**) Total mosquitoes captured across the ten trapping locations per week of the candidate plant host study. (**B**) The proportion of different mosquito species captured across all trapping locations. (**C**) The proportion of males (light green) and females (dark green) captured across the trapping locations (*left*) and the proportion of positive sugar-feeding tests for all males and females. (**D**) The average proportion of positive sugar-feeding tests per week of the field season for males (light green) and females (dark green). (**E**) The average proportion of positive sugar-feeding tests across the field season for each trap site. (**F**) The total number of *Ae. albopictus* captured per week during the candidate plant host study. (**G**) The proportion of positive sugar tests per trap site given by male (light green) and female (dark green) *Ae. albopictus*.

A total of 2360 mosquitoes were collected in Blacksburg, VA, USA, during the field collection season (June-October). The number of collected mosquitoes varied by week, with a large peak occurring in mid-July (Fig. 3A). We identified a total of five species: *Ae. albopictus* (N = 1527; 54.5%), *Culex pipiens* (N = 574; 24.3%), *Anopheles punctipennis* (N = 29; 1.2%), *Ae. vexans* (N = 93; 3.9%), and *Ae. triseriatus* (N = 96; 4.1%) (Fig. 3B). The overall fructose positivity rate was 38% (N = 903 positive tests; Fig. 3C). Throughout the season, this rate generally ranged between 25 and 50% but did not gradually decrease, even towards the end of the season in October (Fig. 3D). In addition, the fructose positivity rate varied across trap sites and ranged between 26 and 46% (Fig. 3E). The majority of mosquitoes captured were females (females: N = 1992; 84%; males: N = 368; 16%). However, we found that males had a higher proportion of positive sugar tests (N = 164; 45%) compared to females (N = 739; 37%) (Fig. 3C). Over 50% of collected mosquitoes were *Ae. albopictus* and their abundance varied by site (Fig. S3A, 3F), and ranged between 40 and 161, with the exception of trap site 8, where the highest number of *Ae. albopictus* mosquitoes were trapped (N = 670; Fig. 3F). The proportion of *Ae. albopictus* testing positive for fructose consumption ranged between 24 and 50% across the ten trap sites (Fig. 3G). (Fig. 3B).

#### Candidate host plants

A total of 75 mosquitoes contained plant DNA that was amplified using the *rbcLa* primers while 144 samples were amplified using *trnH* primers. From these 219 amplifications, we identified 26 unique candidate host plants from 18 different families (Table 2). Interestingly, most of these plants are commonly planted as ornamentals in gardens (*e.g.*, *Prunus* spp.,* Acer *spp.,* Carya illinoinensis*). Eighteen of the 26 candidate host plant sequences were fully assembled; the remaining 8 did not assemble and were identified based on the forward or reverse primer sequences. Most of the plant candidates were identified from DNA that was extracted from *Ae. albopictus* females. However, some were also determined for male *Ae. albopictus* and female *Cx. pipiens*, *An. punctipennis*, *Ae. japonicus* and *Ae. vexans*. Plant species identified from multiple mosquitoes' crops include *Trifolium repens* (*rbcLa, trnH*; N = 12), *Prunus* spp. (*rbcLa*; N = 18)*, Drosera filiformis* (*rbcLa*; N = 2)*, Plantago* spp. (*trnH*; N = 5) and *Acer* spp. (*rbcLa*, *trnH*; N = 4). Most plant species were identified from mosquitoes during specific weeks of the season (Table 2). For example, we found that mosquitoes fed on *Oxalis dillenii* in July and on *Drosera filiformis* only in August. However, species like *Trifolium repens* and *Prunus virginiana* were identified from mosquitoes captured throughout the entire field season. When using *rbcLa* primers, the genus *Prunus* was identified from the crop of both male and female *Ae. albopictus* and female *Cx. pipiens*. For each *Prunus* result, several species consistently appeared with the same high percent identity coverage, indicating that the *rbcLa* gene is highly conserved amongst *Prunus* species. *Prunus* species identified from BLAST present in the study area include *Prunus virginiana* (“bitterberry” or “chokecherry”) and *Prunus padus* (“bird cherry”). *Trifolium repens* was identified frequently throughout the season using both the *rbcLa* and *trnH* barcodes and was targeted almost exclusively by female *Ae. albopictus* mosquitoes.

**Table 2:**
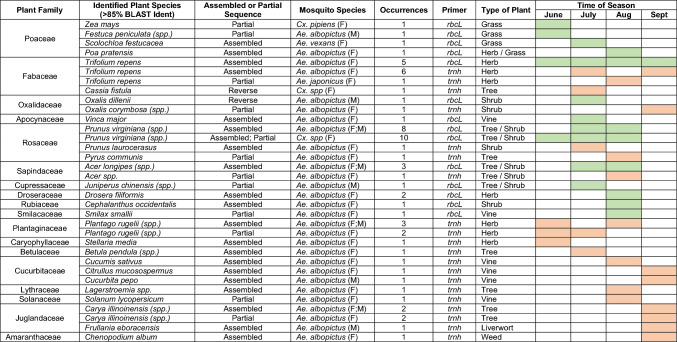
Candidate host plant sources as determined by barcoding and Sanger sequencing.

The species of mosquito that the DNA was extracted from and the number of mosquitoes that contained that DNA is represented. Highlighted boxes on the right indicate when the mosquito fed on the given candidate.

## Discussion and perspectives

The first part of this work revealed that several ornamental plants (8 out of the 11 tested here) can serve as potential sources of carbohydrates to two of the most invasive mosquito species, *Ae. aegypti* and *Ae. albopictus*. Because of the selected plants' short, seasonal blooming period, we could not go beyond testing three individuals of each plant species for each of the mosquito species. While this limits our ability to conclude with confidence about mosquitoes’ preferences between these plant species, it is striking that mosquitoes were attracted to the majority of them. Indeed, the uncertainty around whether the selected individual plants represent the larger population of their species means that, by chance, we could have sampled individuals that were less attractive to mosquitoes. This was not the case, suggesting that the range of plant species that can serve as mosquito hosts is likely larger than previously thought.

The association between the chemical composition of the representative plants we tested and their ability to elicit mosquito attraction and feeding further suggests that mosquitoes can exploit many plants to sustain their metabolism. In addition, this corroborates previous findings [e.g.,^5,26,27^], indicating that combinations of chemicals at specific ratios are more important in explaining mosquito attraction than the simple presence or absence of a given individual chemical. For example, high numbers of landings and feedings on goldenrod were observed for both mosquito species. Occurrences of mosquitoes visiting goldenrod have been recorded as early as 1907^28^, and multiple genera have been observed visiting this plant in the field (personal observations). But the most abundant compound in goldenrod’s scent, *ɑ*-pinene, has been tested against *Ae. aegypti* and *Ae. albopictus* for larvicidal or mosquitocidal effects^29,30^, while its role in mediating attraction or repellency in mosquitoes remains unclear. Similarly, Germacrene D, which has been shown to repel *Ae. albopictus*^31^, was present in high abundance in goldenrod’s scent (15.1%). We observed a similar trend with *Celosia*, marigold, and wave petunia, on which mosquitoes landed despite the presence of *β*-bisabolene in the scent of *Celosia*, which is present in essential oils that repel *Ae. aegypti*^32^. Caryophyllene was the most abundant compound of marigold’s scent and is a major constituent in an essential oil repellent to *Ae. aegypti* but has not been tested with *Ae. albopictus*, potentially inducing higher visitation activity from this species^33^. The scent of yarrow was dominated by cis-Verbenol and *ß*-phellandrene. When mixed with (-)-limonene, *ß*-phellandrene was found to be repellent against *Ae. albopictus* mosquitoes^31^. Cis-verbenol is also present in essential oils that exhibit larvicidal activity against *Ae. albopictus* and *Ae. aegypti*^34^. However, the capacity of these compounds to attract or repel *Ae. aegypti* and *Ae. albopictus* has yet to be tested.

We did not observe feeding activity on wave petunia, yet individuals of both species tested positive for nectar consumption and exhibited the highest carbohydrate concentrations in this study. It is, however, possible that mosquitoes fed on stems/leaves containing sugars either outside of the camera’s frame, or outside of the camera’s recording time at night when petunias emit a stronger scent^1,35,36^. In the headspace of *Lantana*, we identified *β*-ocimene as the most abundant volatile, which has been shown to elicit dose-dependent repellent responses in multiple aedine species, including *Ae. aegypti*^37,38^. Yet, we observed multiple landings from both species. This could be attributed to the ratio of different volatiles in *Lantana’s* scent profile; benzaldehyde was also a major constituent in *Lantana* scent and has been hypothesized to attract mosquitoes when present in volatile blends^39^. In the scent profile of butterfly bush, *ɑ*-farnesene was a prominent volatile; its enantiomer β-farnesene has demonstrated a low degree of repellence in *Ae. aegypti* mosquitoes^40^. However, because mosquitoes have enantioselectivity for odor compounds, *β*-farnesene may not be representative of a response to *α*-farnesene^41^, as implied by the high number of attempted feedings observed from both mosquito species. Vice versa, plants such as *Guara* released high amounts of nonanal, a VOC known to play a role in attracting *Ae. aegypti* to orchids^7^, while seeing the lowest visitation activity from mosquitoes. Finally, *β*-ocimene is a compound with a high degree of ubiquity in plant scents and was the most abundant compound in the scents of Mexican heather (79.24%) and *Scaevola* (56.93%)^42^. Some *Ae. albopictus* were observed feeding on *Scaevola* and tested positive for sugar consumption, but no mosquito was seen feeding on nor tested positive for crop nectar with Mexican heather.

Mosquito attraction to the plants did not necessarily imply successful sugar feeding. Almost 20% of *Ae. albopictus* in plant visitation assays with *Celosia* tested positive for fructose consumption but contained a low concentration of total carbohydrates. Likewise, we observed high landing and feeding activity from *Ae. albopictus* on marigold, but no mosquitoes tested positive for fructose consumption. We also observed high visitation activity on yarrow, which is generally described as mosquito-repellent^43^, but low nectar consumption rates were found in the total carbohydrate assays. This could be attributed to the morphology of *Celosia* (tall, feathery inflorescence) and marigold (wide, bowl-like) flowers not being adapted for a mosquito’s proboscis to probe and feed on. Though yarrow seems to provide a low nutritional reward, it is clear that this flower is not aversive to mosquitoes. Conversely, we observed a low number of feedings from both mosquito species on *Guara*, yet several mosquitoes tested positive for sugar feeding.

Altogether, our plant visitation assays and subsequent chemical analysis suggest that given the broad range of chemical associations present in plant scents, more plants than previously suspected could serve as hosts. Since the chemical analysis supports the idea that mosquito attraction is likely mediated by combinations of chemicals rather than their simple presence/absence, more work is required to fully comprehend the chemical basis of mosquito-plant interactions. In addition, this prompted us to employ a reversed approach and, rather than test each individual plant for mosquito attraction, identify the plants fed upon by local, field-caught mosquitoes.

Using plant DNA barcoding, we identified 26 unique plant species that various local mosquito species, as well as *Ae. albopictus,* feed on in an urban environment and noticed variations depending on the time of year. Overall, the range of plants we identified was broad and encompassed 18 plant families. While most plant species were identified in 1–2 samples, two genera occurred at higher frequencies (in > 12 samples): *Prunus* and *Trifolium*. *Prunus* represents a group of trees and shrubs that are cultivated for their fruits and decorative qualities and *Trifolium* are extremely common in lawns and along roadsides in the United States and produces a substantial amount of nectar in florets that are assumedly shallow enough for a mosquito’s proboscis to reach^44,45^. In a study by Kicel et al.^46^, 1-octen-3-ol was determined as a major constituent in the scent profile of *Trifolium repens*’ stems and leaves. This compound also emanates from human skin and has been reported as a kairomone (*i.e.*, attractant) for *Aedes* mosquito species (*Ae. albopictus* included), especially when acting synergistically with CO_2_^47,48^. Due to the prevalence of *Trifolium repens* in urban locations containing an abundance of hosts, *Aedes* mosquitoes are likely responding to both 1-octen-3-ol and CO_2_ when host-seeking in these areas^49^. In addition, occurrences of *Trifolium repens* have been documented in every continent, and it is able to grow in a range of climates from arctic to tropical, potentially contributing to *Ae. albopictus*’ wide establishment as an invasive disease vector^50,51^.

Another plant host that appeared more than once in our results using both barcodes was the maple tree (*Acer* spp.). Maple trees are some of the most frequently planted ornamental trees in North America^52^. These trees produce flowers and generally bloom from March to June. Interestingly, mosquitoes containing maple tree DNA were captured in July and August. Therefore, it is possible that the captured *Ae. albopictus* mosquitoes obtained carbohydrates by feeding on extra floral nectaries or from the leaves of the maple^1,36^. Alternatively, mosquitoes have been observed to feed on phloem sap, and may be doing so in this case due to the maple tree’s high sap production^53,54^. Due to its commonality in urban settings, maple trees may be contributing to this mosquito’s success in establishing itself as an invasive species.

Mosquitoes have been reported feeding on plant fruits in as early as 1758^55^. Observations of mosquitoes feeding on pears and watermelons have been made in the 1900s, and the data presented here supports this with observed amplifications of *Pyrus communis* (pear) and *Citrullus mucosospermus* (watermelon) DNA^56^. We also identified *Solanum lycopersicum* (tomato), *Cucumis sativus* (cucumber), and *Cucurbita pepo* (pumpkin) as mosquito plant hosts. To our knowledge, this is the first published evidence that *Ae. albopictus*, or any mosquito species may feed on these plants. Furthermore, these plant hosts were identified only from mosquitoes captured in August and September, highlighting the importance of phenological studies for establishing mosquito sugar feeding activity in the wild. Previous studies have shown *An. arabiensis* larvae will feed on the pollen of maize plants in sub-Saharan Africa; gravid female *An. arabiensis* will lay their eggs near it so as to provide food for their offspring^57^. In the present study, *Cx. pipiens* used *Zea mays* (maize) as a plant host, indicating that the attractive blend could potentially be used as a multi-species attractant.

Success rates in species discrimination have been shown to vary between the *rbcLa* and *trnH* barcodes^58,59^, and here we show variations in the plant families they can identify. When comparing the ability of *rbcLa*, *trnH*, and *matK* to accurately identify legume (Fabaceae) species, *trnH* ranked the highest and was only slightly more accurate than *rbcLa*^60^. In the present study, *trnH* identified a higher amount of legume species, but had a similar percent identity to legume DNA sequences amplified by *rbcLa*, supporting the conclusion from Sanchez et al.^60^. A similar study was done previously to compare barcodes in discriminating Cucurbitaceae species, and *trnH* was once again found to exhibit the highest accuracy^61^. The *trnH* barcode identified multiple Cucurbitaceae species in the present study, whereas *rbcLa* identified none. The *rbcLa* barcode identified 18 different DNA sequences belonging to the Rosaceae family and *Prunus* genus but could not discriminate these sequences at the species level.

Overall, this study highlights the importance of expanding our understanding of mosquito phytophagy, particularly for invasive species. We show that *Ae. aegypti* and *Ae. albopictus* can leverage ornamental plants for sugar-feeding and identified a broader than suspected range of plants fed upon by mosquitoes in the wild. We found that mosquitoes can find sugar resources throughout the season which indicate the necessity to further explore their sugar feeding habits in other regions of the US and beyond to better control mosquito populations. Ornamental plants therefore likely help establish mosquitoes’ presence in urban, heavily populated areas. The data presented in this work sets the stage for future systematic surveys of mosquitoes’ host plant selection and could inform the public of plants that may or may not be attracting mosquitoes to the areas they are being planted. In areas with a higher risk of mosquito-borne disease transmission, the experiments described here can be used to enact field removal of plants and to develop baits / repellents to mitigate the spread of disease. Elucidating the mosquito-plant relationship is pivotal for maintaining the development of effective and environmentally friendly disease vector control techniques.

## Materials and methods

### Insects

The Rockefeller *Ae. aegypti* strain (MRA-734, MR4, AATCC®, Manassas, VA, USA) and the ATM95 *Ae. albopictus* strain (ATM-NJ95, AATCC®, Keyport, NJ, USA) were used for this study. These strains are maintained in our laboratory following previously published methods^7^. Larvae were reared in 26 × 35 × 4 cm covered trays that were filled with deionized water. The trays were kept in a climatic chamber at 26° ± 0.5 °C and 60 ± 10% humidity under light:dark cycles of 12 h:12 h. The diet of the larvae consisted of Hikari Tropic First Bites (Petco, San Diego, CA, USA). Prior to starting the plant visitation experiment, around 100 pupae were placed into mosquito breeding containers (BioQuip, Rancho Dominguez, CA, USA—1425, 1425DG) on the day of pupation. Upon emergence, female and male mosquitoes were starved for 1–2 days before being individually selected with forceps. To do so, the containers were placed in a cool environment to immobilize mosquitoes and isolate them for the following plant visitation assays.

### Plant visitation assays

#### Plant visitation protocol

Two BugDorm-1 insect cages (BugDorm, DP1000) were placed on top of a 26 × 35 × 4 cm tray containing DI water to help minimize risks of desiccation. These cages were then placed in a secondary larger acrylic cage (17 × 22 × 32 in) which further provided a warm and humid environment for the mosquitoes. Each assay was conducted with both mosquito species separately to allow for better comparisons between the two species (*e.g.*, survival, feeding). Within each cage, a GoPro camera (Hero5 Black), a water-containing cup covered by a wet paper towel for humidity, and the plant of interest were placed (Fig. 1A). Eleven ornamental plants purchased from local nurseries were tested: wave petunia (*Petunia petunia x atkinsiana*), red impatiens (*Impatiens walleriana*), marigold (*Tagetes* spp.), *Celosia* (spp.), butterfly bush (*Buddleja* spp.), *Guara* (spp.), *Lantana* (spp.), Mexican heather (*Cuphea hyssopifolia*), *Scaevola* (spp.), goldenrod (*Solidago* spp.), and yarrow (*Achillea millefolium*). All methods including plant collection were performed in accordance with the relevant guidelines and regulations. In addition, an iButton (Maxim, DS1923) was programmed and added to the cage to record humidity and temperature throughout the assays. Ten females and ten males of either *Ae. aegypti* or *Ae. albopictus* (3 replicates of 20 mosquitoes per mosquito species using, each time a different individual plant from the same species) were released into different cages. Assays were conducted at the end of the day between 4:30 and 5:30 pm, which has been previously reported as a peak sugar-feeding activity time for *Ae. aegypti* and the beginning of the sugar-feeding rhythm for *Ae. albopictus*^62,63^. In the following morning, between 8:30 and 9:30 am (total: 16 h), alive mosquitoes were collected using a Bug Vacuum (Redeo, XCQ-B), sorted by sex, and stored for subsequent sugar analysis at – 70 ºC. Dead mosquitoes were tallied and removed before beginning another assay to assess survival. Three replicates, totaling thirty mosquitoes per sex per mosquito species, were conducted for each plant species (N = 1320 mosquitoes).

#### Video analysis

GoPro cameras were used to record the mosquito behavior for a duration of two hours. Due to the consistent timing of initializing the assays and beginning the recording, we ensured that the videos captured the peak of sugar-feeding activity in these day-active mosquitoes (between 4:30 p.m. and 7:30 p.m.). Each video was analyzed by counting the total number of mosquito landings and feedings on the flower of interest. Landings were defined as a mosquito flying to the flower and idling on it for any amount of time. A single feeding was defined as the vertical movement of a mosquito’s head in a flower (*i.e.*, probing), which is a characteristic motion of nectar-feeding. The sex of each mosquito landing or feeding was also noted for each landing and feeding event.

#### Data analysis

The effect of the plant species, mosquito species and sex on the number of observed landings and feedings was analyzed by means of Generalized Linear Mixed Models assuming a Poisson error distribution for the number of landings and a quasibinomial error distribution for the proportion of feedings per landing, with a logit link. The replicate number was used as a random effect in the model, and non-significant effects were conserved. The analysis was performed in R (version 4.1.1) using the package lme4 (version 1.1.27.1) and pairwise comparisons were performed using the package emmeans (version 1.7.1.1)^64–67^.

### Scent collection and GC–MS analyses

#### Scent collection

To collect the headspace of each plant species, the inflorescence of the plant was enclosed in a nylon oven bag (Reynolds Kitchens, USA) that was taped tight around the stem. Two tygon tubes (Cole-Parmer, USA) were connected on one side to a diaphragm air pump (Gast, Benton Harbor, MI, USA), while the other side was inserted at the small opening of the bag. Air flow was then initiated by connecting the pump to a 6 V battery (Power-Sonic Batteries, USA). One tube pumped ingoing air into the bag through a charcoal filter cartridge (1 L/min), which served to remove any contaminants from the pump or the surrounding environment. The other tube pulled air out of the bag (1 L/min) through a headspace trap composed of a borosilicate Pasteur pipette (VWR, Radnor, PA, USA) containing 100 mg of Porapak powder Q 80–100 mesh (Waters Corporation, Milford, MA, USA). After 24 h of headspace collection, traps were eluted with 600 μL of 99% purity hexane (Sigma Aldrich, 227064-2L). The samples were sealed in 2 mL amber borosilicate vials (VWR, Radnor, PA) with Teflon-lined caps (VWR, Radnor, PA) and were subsequently stored at − 70 °C to remain stable until analysis by GC–MS. For controls, samples were taken concurrently from empty oven bags as in Lahondère et al.^7^. For each plant species, between 7 and 12 collection replicates were performed using several individuals from the same species.

#### GC–MS analysis

Twenty microliters from each plant scent sample were pipetted from the preceding method and placed into a vial with an insert (VWR, Radnor, PA) to be analyzed with a GC–MS (Thermo Fisher Scientific, Trace 1310) equipped with a 30 m column (Thermo Fisher Scientific, I.D. 0.25 mm, #36096-1420). Helium was used as the carrier gas at a constant flow of 1 cc/min. After each sample was prepared for the species of interest, they were loaded into the machine using an autosampler (TriPlus RSH, Thermo Fisher Scientific). The oven temperature was set at 45 °C, held for 4 min, followed by a heating gradient ramping to 230 °C, held for 6 min (total run: 28.5 min).

Chromatogram peaks were integrated using the Chromeleon software MS quantitative processing method (Thermo Fisher Scientific) and tentatively identified using the online NIST library. Major peaks found with consistently high abundances across multiple samples for each ornamental were then recorded for comparison across ornamental species. An internal standard, made of 100 ng/µL of heptyl acetate (Sigma-Aldrich, CAS #112-06-1), was added to each sample to calculate the concentrations of each compound based on a calibration curve. External synthetic standards, if available commercially, were used to confirm the chemical identity as in Lahondère et al.^7^.

### Carbohydrate content assays

#### Nectar detection in the mosquito crop

Carbohydrate contents were measured by sequentially using the cold and warm anthrone methods described by van Handel^67^. First, mosquitoes were crushed with a glass rod in culture glass tubes (Sigma-Aldrich, C1048-72EA) containing 300 μL of cold anthrone reagent to detect for fructose consumption, as fructose is a monosaccharide that would only be present in the mosquito if it fed on a plant. The anthrone reagent was prepared by combining 150 mL water in a 1 L Erlenmeyer flask on ice with 380 mL sulfuric acid (Fisher, CAS #7664-93-9), in which 750 mg of anthrone (Sigma-Aldrich, CAS #90-44-8) was then dissolved. The samples were kept idle at room temperature (25 °C) for 30 min, after which their color was compared against a negative control (yellow) and positive control (dark green) containing a mosquito which was fed with a 20% fructose solution (Fig. 1D).

#### Quantitative carbohydrate assays

Following this, glass tubes containing the cold anthrone samples were filled with anthrone reagent to a 5 mL mark, heated for 17 min at 92 °C in a dry bath, and then cooled before being vortexed for 15–20 s. A sample without a mosquito was prepared additionally as a control and blank for the spectrophotometer (Perkin Elmer Lambda 20 UV/Visible Spectrophotometer). The optical density (OD) of each sample was then determined at 625 nm. For samples with an OD_625_ above one, 200 μL of sample was diluted with 800 μL anthrone reagent that had been heated as above, giving a dilution factor of 5. The carbohydrate content was quantified using the OD values and a calibration line that had been created by performing the above procedure with samples containing 25, 50, 100, 150, and 200 μg of glucose solution.

#### Data analysis

The effect of the plant species, mosquito species and sex on the number of measured amount of carbohydrates and proportion of positive-testing mosquitoes was analyzed by means of Generalized Linear Models assuming a Gaussian error distribution for the amount of carbohydrates and a binomial error distribution for the proportion of positive-testing mosquitoes per assay. Similarly, the effect of the plant species, mosquito species and sex on the proportion of surviving mosquitoes was analyzed by means of Generalized Linear Models assuming a binomial error distribution for the proportion of survivors per assay. The analysis was performed in R (version 4.1.1) using the package lme4 (version 1.1.27.1) and pairwise comparisons were performed using the package emmeans (version 1.7.1.1)^64–66^.

### Plant DNA barcoding assay

#### Mosquito trapping

Throughout the field season of 2021 (Late May–End of October), mosquitoes were collected weekly from ten residential yards in Blacksburg, VA (USA) containing a high diversity of ornamental and wild plant species. Mosquitoes were captured once a week using BG-2 Sentinel Traps (BioQuip) baited with an attractive lure and carbon dioxide released by dry ice placed in an adjacent cooler (BioQuip). The traps were deployed late afternoon and retrieved mid-morning (total: 16–17 h), which prevented mosquitoes from drying out or digesting crop sugars, which usually occurs within 24 h after ingestion^25^. To further limit sugar digestion and degradation, mosquitoes were transported from the field to the lab on ice and stored at – 70 ºC.

#### Mosquito sample processing and DNA extraction

Mosquitoes collected from the field were identified by morphological traits under a microscope^68^, and washed in phosphate-buffered saline (Sigma-Aldrich, #806552-1L) to remove plant material contaminants, after which they were crushed in a microcentrifuge tube containing 100 μL of 0.3 M sodium acetate (Thermo Scientific, AM9740) and 200 μL of absolute molecular grade ethanol (Sigma-Aldrich, E7023-500 mL) as in Wanjiku et al.^69^. After incubation for 30 min at − 20 °C, homogenates were centrifuged at 4 °C for 10 min at 12,000 g. Following centrifugation, 200 µL of the sample supernatant was tested for the presence of fructose using the cold anthrone method described above. After a 24-h drying period, pellets were extracted using the RED Extract-N-AMP plant DNA extraction kit (Sigma-Aldrich, XNAP-1KT), according to the manufacturer’s instructions. Leaves from *Trifolium repens* collected in the field had their DNA extracted in a similar fashion and were used as a positive control.

#### PCR and Sanger sequencing

DNA was extracted from samples that gave a positive result for the anthrone test and genes of interest were amplified using PCR. Each sample was composed of 200 ng of DNA, PCR grade water (Fisher, AM9935), 10 μL of MyTaqHSmix (Bioline BIO-25045), 0.4 μM of forward and reverse primers targeting the chloroplast ribulose-1,5 biphosphate carboxylase/oxygenase large chain gene (*rbcLa*) (Primer R: GCTTCGGCACAAAAKARGAARCGGTCTC; Primer F: TATGTAGCTTAYCCMTTAGACCTTTTTGAAGA) or *trnh-psbA* intergenic spacer region (*trnH*) (*trnH*: CGCGCATGGTGGATTCACAATCC; *psbA*: GTTATGCATGAACGTAATGCT) to reach a sample volume of 22 μL. PCR cycling conditions for the *rbcLa* barcode were conducted as in Wanjiku et al.^69^ with an initial denaturation of 95 °C for 1 min, 35 cycles of denaturation at 95 °C for 15 s, annealing at 50 °C for 40 s, and extension at 72 °C for 1 min followed by one cycle of final extension at 72 °C for 10 min. For the *trnH* barcode, cycling conditions followed Nyasembe et al.^37^ with a denaturation of 94 °C for 1 min, followed by 45 cycles of denaturation at 94 °C for 1 min, annealing at 55 °C for 40 s and 72 °C for 1 min, and a final extension at 72 °C for 10 min. 1% agarose gels and a 1 Kb DNA ladder (Genesee Scientific, 42-432) were used to visualize and confirm the size of the PCR products. Samples with clear, single bands of the correct size on the agarose gel were sent to Genewiz (South Plainfield, NJ, USA) for purification and Sanger sequencing. Sequences were then assembled using CLC main workbench (Qiagen) and compared to the GenBank database using the Basic Local Alignment Search Tool (BLAST) to search for candidate host plant species; only sequencing results with more than 85% homology were considered as potential candidates.

### Supplementary Information

Supplementary Figure S1.

Supplementary Figure S2.

Supplementary Figure S3.

## Data Availability

Data are available here: https://github.com/mosquito-hub/mosquito_sugar_feeding.git.
