# Supplementary material for: Sugar feeding by invasive mosquito species on ornamental and wild plants
Source: Sci Rep. 2023 Dec 13;13:22121. doi: 10.1038/s41598-023-48089-2 (PMC10719288; doi:10.1038/s41598-023-48089-2)
Supplement: Supplementary file 1 — Supplementary Figure S1. [file 41598_2023_48089_MOESM1_ESM.pdf]

Figure S1

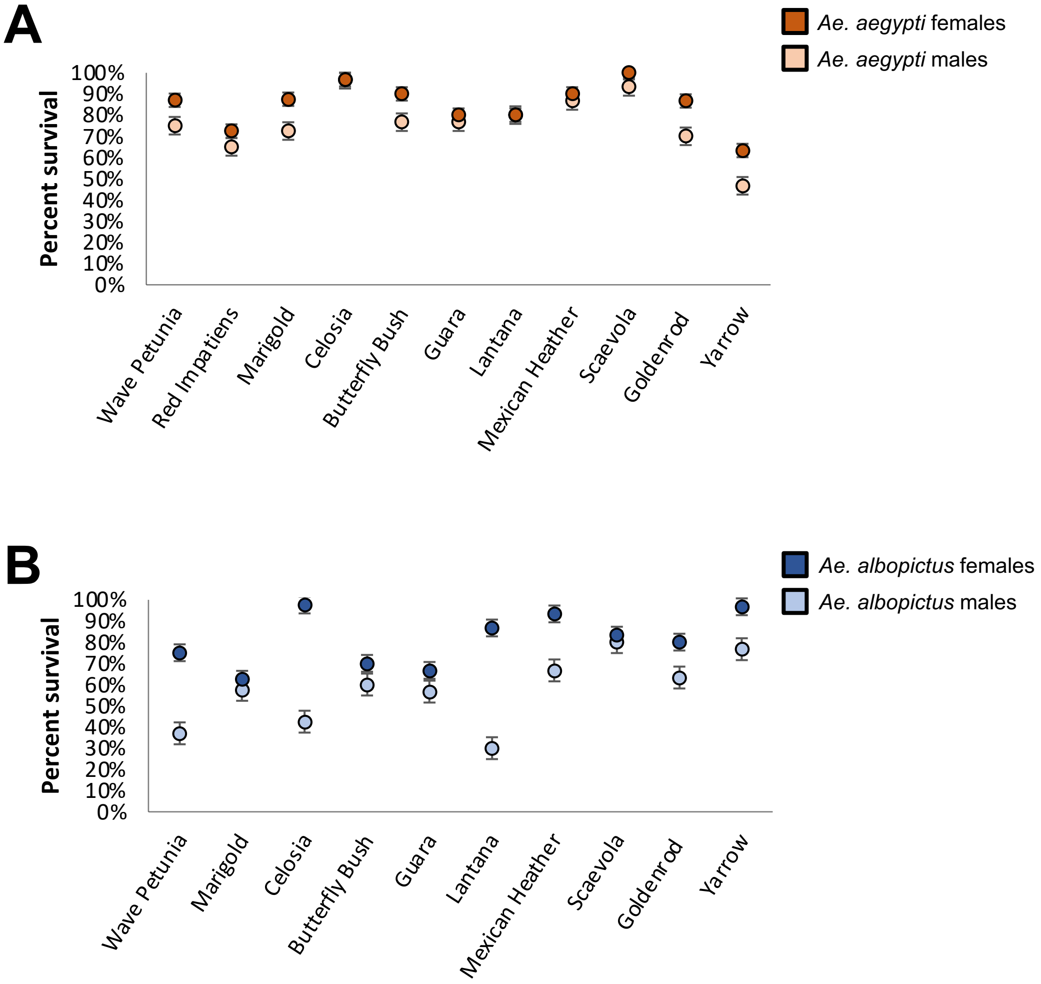

Figure S1. Survival rate of mosquitoes used for plant visitation assays (16 hrs; 9 a.m. - 5 p.m.). Percentages are represented as an average of the three replicates (n = 30 for each data point) performed for each plant species. (A) *Ae. aegypti* survival rate for males (light orange) and females (dark orange). (B) *Ae. albopictus* survival rate for males (light blue) and females (dark blue).
