# Supplementary material for: Sugar feeding by invasive mosquito species on ornamental and wild plants
Source: Sci Rep. 2023 Dec 13;13:22121. doi: 10.1038/s41598-023-48089-2 (PMC10719288; doi:10.1038/s41598-023-48089-2)
Supplement: Supplementary file 2 — Supplementary Figure S2. [file 41598_2023_48089_MOESM2_ESM.pdf]

Figure S2

A

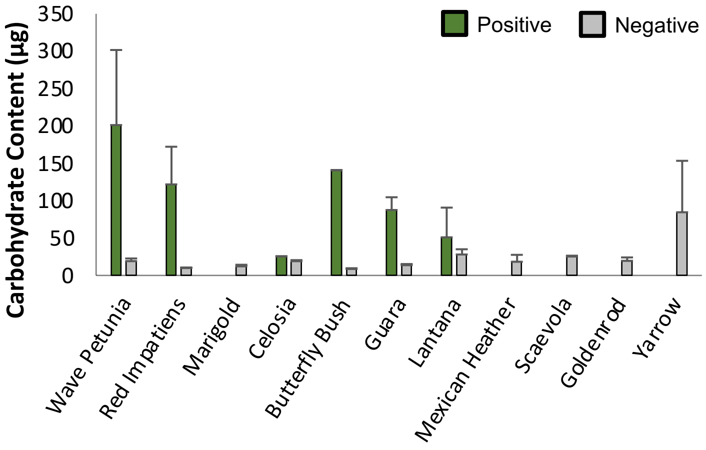

B

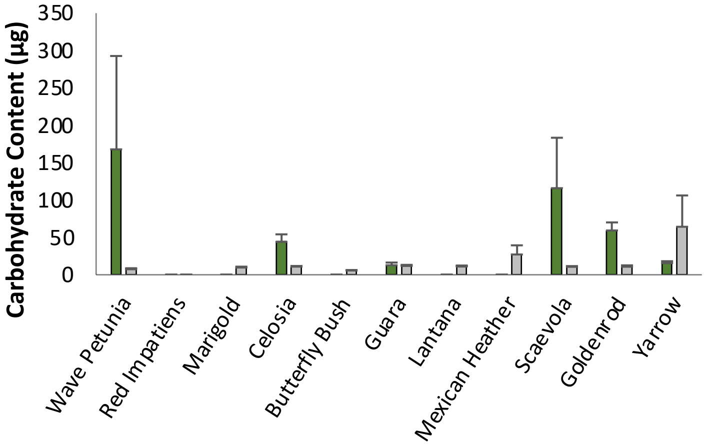

Figure S2. Total carbohydrate concentrations (µg) for (A) fructose-positive mosquitoes. Carbohydrate concentrations for fructose-positive and fructose-negative mosquitoes are also represented for (B) *Ae. aegypti* and (C) *Ae. albopictus*. Values are represented as averages of all mosquitoes that were alive following the plant visitation assays and could be measured for carbohydrates.
