# Supplementary material for: Sugar feeding by invasive mosquito species on ornamental and wild plants
Source: Sci Rep. 2023 Dec 13;13:22121. doi: 10.1038/s41598-023-48089-2 (PMC10719288; doi:10.1038/s41598-023-48089-2)
Supplement: Supplementary file 3 — Supplementary Figure S3. [file 41598_2023_48089_MOESM3_ESM.pdf]

Figure S3

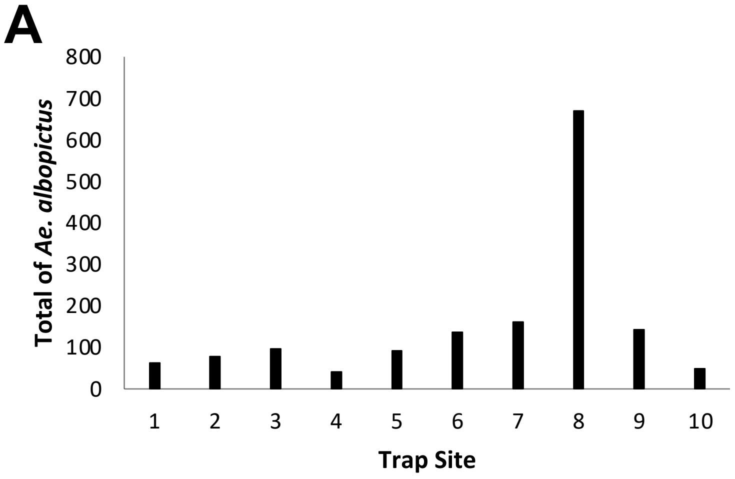

Figure S3. *Aedes albopictus* prevalence and sugar-feeding activity in residential Blacksburg. (A) Total *Ae. albopictus* captured across each of the ten trapping sites.
